# Are Dysregulated Inflammatory Responses to Commensal Bacteria Involved in the Pathogenesis of Hepatobiliary-Pancreatic Autoimmune Disease? An Analysis Using Mice Models of Primary Biliary Cirrhosis and Autoimmune Pancreatitis

**DOI:** 10.5402/2011/513514

**Published:** 2011-06-15

**Authors:** Naoko Yanagisawa, Ikuko Haruta, Ken Kikuchi, Noriyuki Shibata, Junji Yagi

**Affiliations:** ^1^Departments of Infection Control Science and Bacteriology, School of Medicine, Juntendo University, Tokyo 113-8421, Japan Departments of Microbiology and Immunology, Tokyo Women's Medical University, 8-1 Kawada-cho, Shinjuku-ku, Tokyo, Japan; ^2^Department of Infection Control Science, Faculty of Medicine, Juntendo University, Tokyo, Japan; ^3^Department of Pathology, Tokyo Women's Medical University, Tokyo, Japan

## Abstract

The etiopathogenesis of many autoimmune disorders has not been identified. The aim of this paper is to focus on the involvement of bacterial exposure in the pathogenesis of primary biliary cirrhosis (PBC) and autoimmune pancreatitis (AIP), both of which are broadly categorized as autoimmune disorders involving hepatobiliary-pancreatic lesions. Avirulent and/or commensal bacteria, which may have important role(s) as initiating factors in the pathogenesis of autoimmune disorders such as PBC and AIP, will be emphasized.

## 1. Autoimmune Diseases Associated with Microbial Infection

*Autoimmune diseases* arise from an overactive immune response of the body against tissues normally present in the body. Autoimmune disorders are often described as a condition involving genetic components. A high prevalence of autoimmune diseases is observed within families [1]. However, the fact that the prevalence of autoimmune diseases never reaches 100% among monozygotic twins suggests that both genetic and environmental factors are involved in the etiology of autoimmune diseases.

Several studies have implicated microbes in the environmental etiology of autoimmune disorders based on observations such as the regression of autoimmune thrombocytopenia after the eradication of *Helicobacter pylori* [2]. In Guillain-Barré syndrome (GBS), amino acid similarities exist between the gangliosides of the nerve system and the lipopolysaccharides (LPSs) of *Campylobacter jejuni*, suggesting that sensitization by microbes may be based on autoimmunity from molecular mimicry between bacteria and the targeted system of the host [3, 4]. A common recent theory of the cause of autoimmune diseases is that an infectious agent triggers a cycle of events, which leads to the upregulation of the host immune response to self-antigens [5, 6].

## 2. Innate Immunity Sensitized by Bacteria as an Environmental Factor in the Etiology of PBC

Primary biliary cirrhosis (PBC) is a chronic autoimmune disorder characterized by chronic nonsuppurative destructive cholangitis (CNSDC) of small intrahepatic bile ducts and epithelioid granuloma formation in the portal area, which leads to progressive ductopenia [7, 8]. The pathogenesis of biliary epithelial cell damage in PBC is not clearly understood. However, the relationship between bacterial infection and the pathogenesis of PBC through the mechanism of molecular mimicry has become a focus of attention. Evidence of *Propionibacterium acnes* DNA has been detected in the epithelioid granulomas of PBC patients [9]. Sera from patients with PBC have been reported to react with both human and *Escherichia coli *pyruvate dehydrogenase complex E2 (PDC-E2) [10], and such reactivity of antimitochondrial antibodies (AMAs) to both human and bacterial molecules has stimulated speculations that PBC may be induced by exposure to enterobacterial antigens, perhaps by sharing molecular mimicry with mitochondrial antigens in PBC. Other reports indicating bacterial involvement in the etiology of PBC include the reported presence of AMAs, which reacted to *Novosphingobium aromaticivorans* in sera from PBC patients [11], and cross-reactivity detected between *Lactobacillus delbrueckii *beta-galactosidase and PDC-E2 with AMA [12]. A link between toll-like receptors (TLRs) and PBC has also been reported. The elevated expression of TLR4 has been observed in intrahepatic bile duct epithelial cells in PBC [13]. Bacterial components, such as LPSs and the TLR9 ligand CpG DNA, trigger peripheral lymphocytes, monocytes, and bile epithelial cells to produce cytokines and AMAs, suggesting that the pathogenesis of PBC is mediated through TLRs [13–17]. Moreover, elevated levels of immunoglobulin M (IgM) in PBC have been reported to result from the CpG DNA stimulation of TLR signalling, leading to the appearance of IgM-positive memory B cells [18]. 

We previously reported that the Gram-positive bacterial cell wall component lipoteichoic acid (LTA) was detected at the portal tract in the livers of PBC patients with CNSDC and that PBC patients had higher serum IgA class anti-LTA titers, when compared with healthy donors [19]. We analyzed the immunoreactivity against 15 strains from different species of the streptococcal genus using various sera from PBC patients and performed further assays using five strains of the* Streptococcus anginosus* group, the titers of which were higher than those of the other strains, and found that the PBC patient's sera had the highest IgM class titers to *Streptococcus intermedius* [20]. Generally, PBC is characterized by a high serum level of IgM [21]. Our observation may indicate that IgM, in part, is involved in the streptococcal-mediated inflammatory response in PBC. 

*S. intermedius* forms part of the commensal bacteria in the oral cavity, attaching to a surface, forming matrix-enclosed biofilms in dental plaques, and thereby exhibiting increased resistance to antimicrobials and to host immune defense mechanisms [22, 23]. The high immunoreactivity of PBC patient sera against oral streptococci and the high familial prevalence of PBC suggest not only a genetic etiology but also a possible environmental transmission during childhood via the mouth of a child's parent. This theory of transmission from parent to child, however, cannot fully explain the dominant occurrence of PBC in women. 

Considering the fact that the onset of PBC occurs during the fifth decade of life [7] and that there is no apparent antecedent infection related to PBC, as there is in GBS [3, 4], the environmental etiopathology of bacteria in PBC may possibly suggest the chronic persistence of low-immunogenic or avirulent bacteria, commensally coexisting within the host over the long term, rather than a severe yet transient infection. In this sense, periodontitis isolates and other oral colonizing bacteria satisfy these criteria. How do bacteria in the oral cavity reach the liver? One possibility would be a hematogenous transmission from the oral mucosal layer. Since increased permeability of the stomach and the small intestine is reported in PBC patients [24], transmission from the intestinal tract via the portal vein to the liver should also be an additional possibility.

## 3. Infection-Induced Mouse Model of PBC

Whereas strong evidence exists that bacterial infection may trigger PBC, subsequent implications have been difficult to elucidate. Oral streptococcal isolates, namely, *S. intermedius*, *S. sanguinis*, and *Streptococcus mitis *as well as *Micrococcus luteus* and *E. coli*, were repeatedly used to inoculate BALB/c mice, mimicking chronic bacterial exposure, and pathological alterations in the liver and the production of autoantibodies were investigated. The livers of mice at 8 weeks after inoculation with *S. intermedius*, *S. sanguinis*, or *S. mitis* showed CNSDC-like inflammatory cellular infiltrates in the portal area [25]. When BALB/c mice were left inoculation-free for an additional 20 months after the completion of an initial 8 week-*S*.* intermedius* inoculation, CNSDC-like portal inflammation was still observed [25]. LTA immunoreactivity was detectable in not all but some of the cytoplasm of polymorphic inflammatory cells around biliary epithelial cells and connective tissues around bile ducts, and CD3-positive cells were predominantly observed in the cellular infiltrates around the bile ducts. In the portal area, LTA immunoreactivity was detectable in not all but some of the cytoplasm of polymorphic inflammatory cells around the biliary epithelial cells and connective tissues around the bile ducts, and CD3-positive cells were predominantly observed in the cellular infiltrates around the bile ducts. Other tissue damages that are occasionally associated with PBC were also detected. The salivary glands of live *S*.* intermedius*-inoculated mice exhibited Sjögren's syndrome-like periductal lymphocytic infiltration [25]. 

An investigation of PBC-like autoantibodies in our model detected a high frequency of the production of anti-gp210 antibodies, which are commonly detected in PBC patients, especially those with a poor prognosis [26]. We previously reported that IgM class antibody titers against histone-like DNA-binding protein (HLP) of *S. intermedius* were significantly high in the sera of PBC patients and that immunoreactivity to anti-*S. intermedius*-HLP was detected in the cytoplasm of biliary epithelial cells and inflammatory cells in the portal area of the livers of PBC patients [20]. In *S. intermedius*-inoculated BALB/c mice, consistent with observations in human patients, immunoreactivity to *S. intermedius*-HLP was detected around the sites of CNSDC in the portal area of the livers both soon after and 8 weeks after *S. intermedius* inoculation. Furthermore, the affinity of anti-*S. intermedius*-HLP antibody to the c-terminus synthetic peptide of gp210 was detected in a dose-dependent and specific manner, suggesting cross-reactivity between gp210 and *S. intermedius*-HLP [25]. These results suggested that chronic exposure to *S. intermedius* could trigger PBC-like pathological alterations in BALB/c mice resembling the pathology of PBC in humans.

## 4. Autoimmune Pancreatitis and IgG4-Related Diseases

Autoimmune pancreatitis (AIP) is another putative autoimmune disease of the hepatobiliary-pancreatic system and is a chronically progressing inflammatory disease of the pancreas [27, 28]. The morphological characteristics of AIP include diffuse or localized enlargement of the pancreas and irregular narrowing of the main pancreatic duct. Histologically, the disease is also associated with progressive lymphoplasmacytic infiltration, predominantly localized to the ductal structures, and varying degrees of parenchymal and acinar destruction [29]. 

There are two types of AIP that differ in their clinical features, such as the gender ratio, mean age, and associated immune-related diseases. Type 1 AIP is associated with the histological finding of lymphoplasmacytic sclerosing pancreatitis (LPSP). Its serological hallmark is an elevation in the serum levels of the IgG4 subclass of IgG [27]. Type 1 AIP appears to be the pancreatic manifestation of a systemic disease called IgG4-associated systemic disease (ISD), affecting not only the pancreas but also other organs including the bile duct, retroperitoneum, kidney, lymph nodes, and salivary glands [30]. Type 2 AIP is a form of idiopathic chronic pancreatitis, histologically associated with granulocyte-epithelial lesions [31]. The pathogenesis of AIP remains unknown. Genetic associations between susceptibility to the disease and the human leukocyte antigen (HLA) DRB1*0405-DQB1*0401 haplotype [32–34], Fc receptor-like gene 3 (FCRL3) [35], and the CTLA4 gene [36] have been suggested. 

An outstanding finding in type 1 AIP is hypergammaglobulinemia and the existence of a high serum concentration for IgG4, which has been documented in 90% of patients [37]. This occurs in parallel to an abundant IgG4-positive plasma cell infiltration in the pancreatic tissue [38]. The fibroinflammatory process characterizing AIP occurs at the pancreatic basement membranes where IgG4/IgG/complement-immune complexes are deposited [39]. AIP is occasionally associated with elevated circulating immune complex levels, which are significantly linked to increased serum IgG1 and complement activation via the classical pathway [33]. IgG4 is unable to activate the classical pathway of complements but binds IgG1, 2, and 3 and forms an Fc–Fc interaction immune complex in patients with AIP [40]. Although the role of IgG4 in the immune response and autoimmunity has not yet been fully elucidated, it may be hypothesized that IgG4 blocks the Fc-mediated effector functions of IgG1 and dampens the inflammatory response to an as-yet-unidentified primary trigger of the inflammatory process in AIP [40, 41].

## 5. Autoantibodies in AIP

To which antigens the immunoglobulins in the deposits along the basal membranes of the pancreatic ducts and acini react remains unclear. Circulating antibodies in AIP include autoantibodies against carbonic anhydrase- (CA-) II [62], CA-IV [63], lactoferrin (LF) [62], pancreatic secretary trypsin inhibitor (PSTI) [64], and heat shock protein- (HSP-) 10 [65]. Thymectomized mice immunized with CA-II or LF develop a pathology that closely resembles AIP under a regulatory T cell- (Treg-) depleted background [54]. However, T cells specific for CA-II and LF were unable to induce pancreatitis in the adoptive transfer of an amylase-specific rat model [52], implying that autoantibodies against these enzymes in AIP represent a late consequence of tissue destruction and perhaps not a fundamental pathogenic mechanism. Additionally, these potential antigens reside in the cytoplasm of pancreatic cells and are not associated with the basement membranes targeted for the fibroinflammatory process in AIP [39]. Moreover, the major subclasses of these autoantibodies in the sera of patients with AIP are classified as the IgG1 subtype, and not IgG4 [33, 40, 64]. This implies that these antigens may be secondarily involved in the AIP immune process, as it is directed toward still-unknown antigens in pancreatic basal membranes.

## 6. Innate Immunity Sensitized by Infectious Agents in the Etiology of AIP

Although the initial events triggering AIP are not known, the pancreatic cells may become targets of immune-mediated processes through viral or other infectious agents. Guarneri et al. showed a significant homology between human CA-II and *α*-CA of *H. pylori*. Moreover, the homologous segments contained the binding motif of DRB1*0405 [66]. Notably, the possession of the HLA DRB1*0405-DQB1*0401 genotype confers a risk for AIP development [32]. Frulloni et al. reported that 94% of AIP patients, but only 5% of pancreatic cancer patients, exhibit IgG antibodies to a plasminogen-binding protein (PBP) that is homologous to the human protein ubiquitin-protein ligase E3 component n-recognin 2 (UBR2), which is expressed in pancreatic acinar cells and is also homologous to the PBP of *H. pylori* [67]. These data suggest that *H. pylori* infection may trigger AIP in genetically predisposed subjects through autoimmune responses triggered by molecular mimicry.

Several experimental models of AIP have been described (Table 1). Virus-induced AIP models, such as C57BL/6 mice infected with the murine leukemia retrovirus LP-BM5, developed histological findings similar to human AIP [59, 60]. The spontaneous development of pancreatitis via an autoimmune mechanism in MRL/Mp mice is accelerated by the administration of polyinosinic : polycytidylic acid (poly I:C), a synthetic double-stranded RNA and TLR3 ligand [55–58]. Sensitization occurs with not only viral components, such as double-stranded RNA poly I:C, but also bacterial LPS-induced pancreatitis in interleukin- (IL-) 10-deficient mice [55]. TLRs play important roles in innate immunity and initiate intracellular signaling to macrophages and dendritic cells after stimulation with various antigens. The majority of known TLRs mediate the development of Th1 cell-inducing dendritic cells [68]. Hence, pattern-recognition receptors (PRRs) that bind pathogen-associated molecular patterns (PAMPs) may trigger an autoimmune response.

## 7. Infection-Induced Mouse Model of AIP

We previously reported that when C57BL/6 mice were inoculated intraperitoneally (i.p.) with heat-killed *E. coli *weekly for 8 weeks, marked cellular infiltration with fibrosis was observed in the exocrine pancreas accompanied by a high serum gamma-globulin level and the production of autoantibodies against CA-II and LF. Bacterial infection apparently triggered autoimmune pancreatitis-like pathological alterations in mice that strikingly resembled AIP in humans [61]. 

C57BL/6 mice inoculated weekly with *E. coli *for 8 weeks were utilized as donors, and the spleens were intravenously transferred to RAG2^−/−^ mice. The pancreas in the recipient RAG2^−/−^ mice showed cellular infiltration in the exocrine pancreas, especially around the pancreatic ducts, indicating that the *E. coli*-inoculated mouse spleen cells possess the ability to reproduce pathological alterations in the pancreas of naïve mice. Similarly, when the spleen cells of donor *S. intermedius*-inoculated mice were transferred to RAG2^−/−^ mice, CNSDC-like cholangitis in the liver was induced, similar to that seen in the donor [61]. 

The AIP-like inflammatory region in the pancreases of recipient mice with spleen cells transferred from *E. coli*-inoculated mice [61], as well as the CNSDC-like inflammatory region in the livers of recipient mice with spleen cells transferred from *S. intermedius*-inoculated mice [25], both showed that most of the cellular infiltrates in the target organs were CD3 positive, indicating that these cells in both models originated from the donor mice. The findings observed indicated that our animal models of PBC and AIP are of autoimmune etiology.

The criteria for determining whether a condition may be considered to be autoimmune, according to Witebsky's postulates with modern revision by Rose and Bona [69], include (i) indirect evidence based on the reproduction of the autoimmune disease in experimental animals, (ii) direct evidence of the transfer of pathogenic antibodies, or (iii) pathogenic T cells and indirect evidence of the isolation of autoantibodies or autoreactive T cells. Several lines of evidence have established our animal models to be of autoimmune origin. The current approach in our research is to search for a possible supply of antigenic stimulant that is similar to or that will cross-react with autoantigens *in vivo*; so far we have narrowed our search down to bacterial species possessing a candidate epitope.

## 8. Conclusions

**Figure 1:**
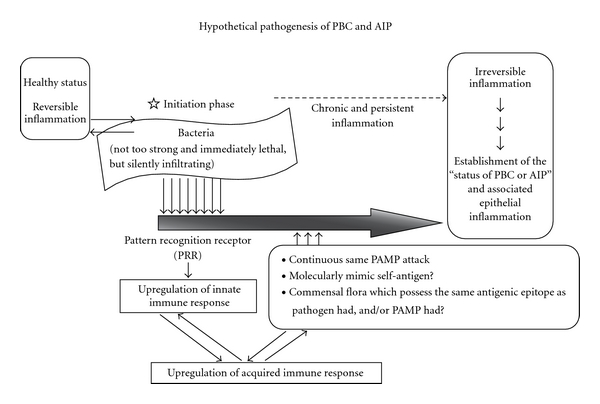
Hypothetical pathogenesis of PBC and AIP. During the initiation phase, weak but silently infiltrating PAMPs and/or antigen(s), such as avirulent bacteria, trigger and upregulate the innate immune system. Second, the progressive phase features the persistence of this PAMP attack or stimulation by molecular mimicry and/or exposure or stimulation from commensal flora possessing the same antigenic epitope that the initial pathogen and/or PAMP possessed, thereby upregulating the host immune response to the target antigen. These slowly progressive steps eventually lead to the development of autoimmune diseases. Modified from previous papers [25, 61].

We propose a hypothetical pathogenetic mechanism for bacteria-induced PBC and AIP. During the initiation phase, weak but silently infiltrating PAMPs and/or antigen(s), such as avirulent bacteria, trigger and upregulate the innate immune system. Second, the progressive phase features the persistence of this PAMP attack or stimulation by molecular mimicry and/or exposure or stimulation from commensal flora possessing the same antigenic epitope that the initial pathogen and/or PAMP possessed, thereby upregulating the host immune response to the target antigen. These slowly progressive steps eventually lead to the development of autoimmune diseases (Figure 1).

Recently, NOD.c3c4 [70], TGFb receptor II dominant-negative [71], IL-2 receptor *α* knockout [72], and* Ae2_a,b_*-deficient mice [73] have been reported as spontaneous PBC animal models. The spontaneous development of AIP in T-cell-competent HLA-DR*0405 transgenic Ab0 NOD mice [49], Treg-deficient backgrounds in neonatally thymectomized mice [54], NOD.CD28 knockout mice [51], and Tgfbr2fsp knockout mice [50] have demonstrated genetic polymorphisms of the effector cells in the etiologies of AIP. However, as these mice are genetically engineered, they may not completely reflect the onset of human diseases. Our models of PBC and AIP developed in genetically normal BALB/c and C57BL/6 mice, respectively, by the inoculation of human commensal bacteria are more clinically relevant, as they reflect the conditions of human patients, and should be useful for advancing our understanding of the pathological mechanism(s) underlying PBC and AIP. 

Under normal conditions, commensal bacteria are not pathogenic and in fact may inhabit the host from early life. Commensal bacteria in the gastrointestinal tract have been recognized to interact with the innate immune system and to drive regulatory T-cell differentiation [74, 75]. The fact that commensal bacteria can induce autoimmune diseases associated with genetic polymorphisms, immune susceptibility, and other environmental factors may expand our concept of the pathoetiology of autoimmune diseases.

## Figures and Tables

**Table 1:** Experimental animal models of autoimmune pancreatitis.

Animal	Organs with lesions	Induction	Target antigen	Effector cells	References
MRL/lpr mice	Pancreas	Spontaneous	?	T cells	[42]
	Pancreas, salivary gland				[43]
*aly/aly* mice	Pancreas, salivary gland, lung	Spontaneous	?	CD4^+^ T cells	[44]
	Pancreas				[45, 46]
WBN/Kob rats, male	Pancreas, salivary gland, bile duct	Spontaneous	?	CD8^+^ T cells	[47]
MHC-II^−/−^ mice	Pancreas	Spontaneous	?	CD8^+^ T cells	[48]
T-cell+ HLADR*0405Ab0 NOD mice	Pancreas, lung	Spontaneous	?	T cells	[49]
Tgfbr2*^(fspKO)^* mice	Pancreas, salivary gland	Spontaneous	?	T cells	[50]
NOD.CD28KO mice	Pancreas	Spontaneous	Amylase	CD4^+^ T cells	[51]
DA(RP) rats Lewis rats	? Pancreas	Amylase-specific T cell	?	CD4^+^ and CD8^+^ T cells	[52]
PL/J mice (H-2^s^, H-2^u^)	Pancreas, salivary gland, kidney	CA-II	CA-II	?	[53]
nTx-BALBc mice	Pancreas, salivary gland, bile duct, kidney	CA-II	CA-II	CD4^+^ Th1 cells	[54]
nTx-BALBc mice	Pancreas, salivary gland, bile duct, kidney	LF	LF	CD4^+^ Th1 cells	[54]
MRL/Mp, MRL/lpr mice	Pancreas, salivary gland, bile duct, kidney		?	CD4^+^ T cells	[57]
MRL/Mp mice	Pancreas, salivary gland, liver	Poly I:C	PSTI	?	[58]
	Pancreas		?	?	[55]
IL-10KO mice	Pancreas	Poly I:C, LPS	?	?	[55]
C57BL/6 mice	Pancreas, salivary gland, liver, kidney, lung	LP-BM5	?	CD4^+^ T cells	[59, 60]
C57BL/6 mice	Pancreas, salivary gland	*E. coli*	?	T cells	[61]

CA-II: carbonic anhydrase II; LF: lactoferrin; PSTI: pancreatic secretary trypsin inhibitor; poly I : C: polyinosinic polycytidylic acid; nTx: neonatal thymectomy.

Modified from [28, 29].

## Abbreviations

AIP: Autoimmune pancreatitis
AMA: Antimitochondrial antibody
ANA: Antinuclear antibody
CA-II: Carbonic anhydrase II
CTLA4: Cytotoxic T-lymphocyte antigen 4
FCRL3: Fc receptor-like gene 3
GEL: Granulocyte epithelial lesions
GBS;: Guillain-Barré syndrome
HLA: Human leukocyte antigen
HSP: Heat shock protein
HLP: Histone-like DNA-binding protein
IDCP: Idiopathic duct-centric pancreatitis
LF: Lactoferrin
LPS: Lipopolysaccharide
LPSP: Lymphoplasmacytic sclerosing pancreatitis
LTA: Lipoteichoic acid
PAMP: Pathogen-associated molecular pattern
PRR: Pattern recognition receptor
poly I:C: Polyinosinic : polycytidylic acid
PBC: Primary biliary cirrhosis
PBP: Plasminogen-binding protein
Treg: Regulatory T cell
TLR: Toll-like receptor
TGF: Transforming growth factor
UBR2: Ubiquitin-protein ligase E3 component N-recognin 2.
